# *CNTNAP1* Mutations and Their Clinical Presentations: New Case Report and Systematic Review

**DOI:** 10.1155/2020/8795607

**Published:** 2020-04-13

**Authors:** Sandra Sabbagh, Stephanie Antoun, André Mégarbané

**Affiliations:** ^1^Pediatrics Department, Hôtel-Dieu de France, Beirut, Lebanon; ^2^School of Medicine, Lebanese University, Beirut, Lebanon; ^3^Institut Jérôme Lejeune, CRB BioJeL, Paris, France

## Abstract

Lethal congenital contracture syndrome type 7 (LCCS7) and congenital hypomyelinating neuropathy type 3 (CHN3) are rare autosomal recessive diseases, characterized by severe neonatal hypotonia, polyhydramnios, arthrogryposis, facial diplegia, and severe motor paralysis, leading to death in early infancy. They are related to mutations in the *CNTNAP1* (contactin associated protein 1) gene, playing an important role in myelination. Recent studies have shown that both diseases could present with a wide phenotypic spectrum, with promising survival up to early childhood. We report on a 7-year-old boy from a nonconsanguineous Lebanese family presenting with neonatal hypotonia, respiratory distress, and arthrogryposis. Molecular analysis revealed the presence of a pathogenic variant in the *CNTNAP1* gene leading to a premature stop codon: NM_003632.2:c.3361C>T p.(Arg1121^*∗*^). A review of the literature is discussed.

## 1. Introduction

Mutations the *CNTNAP1* gene (contactin associated protein 1) (OMIM 602346) have been associated with two rare autosomal recessive congenital diseases, lethal congenital contracture syndrome type 7 (LCCS7) (OMIM 616286), and congenital hypomyelinating neuropathy type 3 (CHN3) (OMIM 618186). *CNTNAP1* is located on chromosome 17q21 and encodes a contactin-associated transmembrane receptor (CASPR), highly essential in the formation of paranodal axoglial junctions in myelinated axons [1–6].

*CNTNAP1* is also involved in regulating neural progenitor cells and the development of cerebral cortex [1]. It was first implicated in human disease in 2014, leading to disorders characterized by polyhydramnios, severe neonatal hypotonia, arthrogryposis, facial diplegia, and severe motor paralysis, leading to death in early infancy [6]. These mutations have been reported in 31 patients, with a wide phenotypic spectrum and promising survival up to early childhood [7–9].

Here, we report on a new case presenting with LCCS7 with a novel identified *CNTNAP1* variant, with survival beyond early infancy.

## 2. Materials and Methods

This study is conformed to the tenets of the Declaration of Helsinki.

### 2.1. Clinical Presentation

The patient is a 7-year-old boy, born to Lebanese unrelated healthy parents. He is the second of three children, his siblings being both healthy.

### 2.2. Whole Exome Sequencing

After informed consent was obtained by the parents in compliance with national ethics regulation, genomic DNA extraction was performed from peripheral blood using standard techniques.

Approximately 60 Mb of the consensus coding sequences (>99%) were enriched from fragmented genomic DNA using the SureSelect Human All Exon V6 kit (Agilent Technologies, Santa Clara, CA, USA) and the generated library was sequenced on an Illumina HiSeq 4000 platform (Illumina, San Diego, CA, USA) with an average coverage depth of 100x. An end-to-end bioinformatics pipelines including base calling, primary filtering of low quality reads and probable artefacts, and annotation of variants was applied.

## 3. Results

### 3.1. Clinical Report

Pregnancy was complicated with placental abruption and unexplained polyhydramnios. The patient was born after an emergent C-section at 35 weeks of gestational age, with generalized hypotonia and a severe respiratory distress. His weight was 2340 g (25^th^ percentile), his length was 47 cm (50^th^ percentile), and his head circumference was 36 cm (>97^th^ percentile). On initial examination, he was comatose, hypotonic, with absence of spontaneous motility, and intermittent trepidation of lower limbs. He was intubated and transferred to the neonatal intensive care unit (NICU). Upon admission, initial metabolic and infectious workups were within normal ranges. Cardiac evaluation was normal. The brain magnetic resonance imaging (MRI) showed a slowing of flow in some superficial cortical veins with asymmetric flow in both transverse sinuses with no visible thrombophlebitis. An electroencephalogram monitoring revealed a slowed cerebral activity with intermittent burst suppressions. Neurological workout included the dosage of neurotransmitters in the cerebrospinal fluid (CSF) that identified a dosage of L-dopa at 31 nmol/L (reference range lower than 30 nmol/L), a low dosage of homovanillic acid (HVA) (<543 nmol/L; reference range between 601 and 1397 nmol/L), and a low dosage of 5-hydroxy-indole acetic acid (5-HIAA) (<383 nmol/L; reference range between 382 and 949 nmol/L).

On clinical examination at one month of age, the patient remained hypotonic, areflexic, with no visual tracking nor blinking, and an absence of muscular movements of the intercostal muscles. He also presented a major plagiocephaly and a persistent upper limbs contraction. He was noted to have epicanthal folds, thickened nares, and thickened lips. He received valproic acid for recurrent generalized clonic movements. Dilated fundus examination revealed an anterior subcapsular cataract in the right eye. The patient underwent tracheotomy and gastrostomy. Complementary metabolic workout was within normal ranges, including amino acid and organic acid chromatography. Muscle biopsy revealed muscular hypertrophy with mitochrondrial and neurogenic dysfunction. Congenital disorders of glycosylation were ruled out, as well as Prader-Willi syndrome, by genetic studies. Finally, a tracheotomy was undergone because of the impossibility of ventilation weaning, and the patient was fed by gastrostomy, due to the absence of swallowing.

Afterwards, no changes occurred in his clinical status. At seven years, generalized hypotonia remained prominent, and the patient showed a failure to thrive, with a weight of 16 kg (<1^st^ percentile), a length of 102 cm (<1^st^ percentile), and a head circumference of 56 cm (>97^th^ percentile). Tracheotomy weaning turned out to be impossible and nutrition was provided exclusively by gastrostomy. No infections were detected and the patient received all his immunizations.

### 3.2. Molecular Analysis

By whole-exome sequencing (WES), we detected a homozygous variant in the *CNTNAP1* gene NM_003632.2:c.3361C>T p.(Arg1121^*∗*^) that creates a premature stop codon. This variant has been confirmed by Sanger sequencing and found at the heterozygous state in both parents. It was classified as likely pathogenic (class 2) according to the recommendations of the American College of Medical Genetics.

## 4. Discussion

*CNTNAP1* involvement in human diseases was first described by Laquérriere in 2014 [6]. To this day, most of the 31 identified patients (11 LCCS7 and 20 CHN3), issued from 20 families, were males and answered to diverse ethnicities (Palestinian, Irish, English, American, Qatari, Lebanese, and French), which brings the number of affected families to twenty [1, 2, 6–11]. Only seven girls were reported to be affected, three of which were diagnosed with LCCS7 and four with CHN3 [8–10]. First degree consanguinity was noted in most families [6–8, 10].

### 4.1. Clinical Features

All affected patients presented with respiratory distress at birth, secondary to motor nerve paralysis. Most of them were born preterm (58%) and had marked fetal akinesia (42%) with polyhydramnios (97%). Another classical finding was the absence of swallowing (58%). However, arthrogryposis multiplex congenital was present in 42%, with a nonspecific joint involvement. Hypotonia (87%) remains frequent in this population. Other findings remain rare and nonpathognomonic (Table 1).

The above described patient answers to most of the clinical features reported previously. He also presented some of the rare features cited in the literature, such as seizures and epicanthus. To this date, this is the only surviving patient showing evidence of an isolated unilateral anterior subcapsular cataract in association with LCCS7. No known cause of juvenile nor congenital cataract was identified. The ophthalmological examination was otherwise within normal ranges, and the seizures were well controlled under treatment. Moreover, no other reported case underwent a dosage of CSF neurotransmitters, which could be a track to follow for further studies of management.

### 4.2. Histopathology

Nerve biopsy is now less often performed, as genetic testing in patients with hereditary neuropathies is becoming increasingly used [7, 12]. However, sural nerve biopsy is nonspecific and consists mainly of paranodal abnormalities [7, 9], along with significant homogeneous axonal loss [1, 6, 9, 11], significant decrease of large myelinated fibers [1, 10, 11] and thin myelin sheaths [1, 6, 9–11], all consistent with hypomyelinating neuropathies [1, 7, 10]. Muscle biopsy can reveal early muscle innervation disorder [11] with generalized small muscle fibers [8, 9] and neurogenic muscular atrophy [8, 9]. On electron microscopic examination, a marked widening of the nodes of Ranvier is often noticed [1, 2, 6, 11], leading to a loss of attachment sites of the myelin loops in the paranodal regions [1, 2, 11] and an absence of the typical transverse bands that cross the paranodal junctional gap [1, 13].

### 4.3. Imaging

Magnetic Resonance Imaging (MRI) is also nonspecific and could reveal signs of hypomyelination [1, 7–10] and cerebral atrophy [1, 8–10]. Cases have been reported with normal brain MRI [1, 2, 8, 11].

### 4.4. Functional Explorations

Electroencephalography (EEG) might show a discontinuous immature pattern [7], while electromyography (EMG) and nerve conduction studies converge to a neurogenic rather than a myopathic pattern [7, 9]. Auditory and visual evoked potentials could be nonspecifically altered [11].

### 4.5. Antenatal Diagnosis

Fetal sonography can detect prenatal manifestations for LCCS7, such as fetal hypokinesia, abnormal positioning of the limbs, and polyhydramnios that require therapeutic amniotic fluid reductions [1, 8]. No interference with fetal growth velocity has been reported [8]. Detection can go as far as 27 weeks of gestation [1, 8]. No patient has been recorded to be born before 30 weeks of gestation [10].

### 4.6. Genetic Counseling

The present case identifies a new pathogenic variant in the *CNTNAP1* gene, leading to a premature codon stop. The list of reported variants is presented in Table 2. While CHN3 can be caused by either homozygous or compound heterozygous mutation in the *CNTNAP1* gene, LCCS7 is only caused by a homozygous mutation.

### 4.7. Management

Although there is no evidence of a milder phenotype, extensive medical care might explain the survival of twelve cases to this day [1]. Of the latter, four underwent gastrostomy and twelve tracheostomy at the neonatal intensive care unit [1, 8–10]. Supportive treatment must be provided accordingly.

### 4.8. Prognosis

Life expectancy in patients with LCCS7 is usually limited to death in the neonatal period, varying from a few hours of life to three months [1, 2, 4, 6, 8, 10, 11]. However, patients with CHN3 are reaching early teenager years, with the oldest reported patient at 15 years old [8]. To this day, ten patients with CHN3 are reported to be alive, and only one girl with LCCS7 [7–9]; we report on the second LCCS7 patient to remain alive, leading to a total of twelve cases. Respiratory distress was the main cause of death.

### 4.9. Differential Diagnosis

CHN were first described by Dejerine and Sottas in the late 1800s, and they consisted essentially of progressive muscle wasting [10]. Later on, a larger spectrum has been identified and new classifications emerged [1, 7, 14, 15]. CHN present with hypotonia, areflexia, and distal muscle weakness [15–19]. CHN1 (OMIM 605253) has commonly been linked to mutations in *EGR2* gene, while CHN2 (OMIM 618184) is associated with mutations in *MPZ* gene [10, 11]. Interestingly, no intellectual disability was reported in these two types, in opposition to CHN3 [1, 2, 11]. Nerve histology remains nonspecific in CHN with severe hypomyelination, whereas other hypomyelinating diseases, such as Dejerine-Sottas Syndrome or Charcot-Marie-Tooth type 1, a redundant “onion bulb” formation is found [7, 20, 21].

Arthrogryposis multiplex congenita (AMC) has been linked to fetal hypokinesia and underlying neurologic disease [22]. It can result from an impairment of skeletal muscles, or the neuromuscular junction or axoglial development [6, 22]. However, it can also be caused by nongenetic factors, namely maternal autoimmune myasthenia or mechanical limitations of fetal movements [6]. As recorded by Hengel et al. in 2017, AMC is no longer an obligate feature of hypomyelinating neuropathies and could either be the primary manifestation of a musculoskeletal disease or the secondary manifestation of a neurologic process [1]. Consequently, in opposition to LCCS7, CHN3 is not associated with AMC [1].

Moreover, LCCS form a heterogeneous family of 11 types that were all initially characterized by hypokinesia, AMC, and early neonatal death [23]. All types have been linked to non-overlapping genetic mutations: LCCS1 (OMIM 253310) caused by mutation in the *GLE1* gene, LCCS2 (OMIM 607598) caused by mutation in the *ERBB3* gene, LCCS3 (OMIM 611369) caused by mutation in the *PIP5K1C* gene, LCCS4 (OMIM 614915) caused by mutation in the *MYBPC1* gene, LCCS5 (OMIM 615368) caused by mutation in the *DNM2* gene, LCCS6 (OMIM 616248) caused by mutation in the *ZBTB42* gene, LCCS8 (OMIM 616287) caused by mutation in the *ADCY6* gene, LCCS9 (OMIM 616503) caused by mutation in the *ADGRG6* gene, LCCS10 (OMIM 617022) caused by mutation in the *NEK9* gene, and LCCS11 (OMIM 617194) caused by mutation in the *GLDN* gene.

## 5. Conclusion

In conclusion, LCCS7 and CHN3 are rare disorders, associated to a *CNTNAP1* mutation. It combines severe neonatal hypotonia, to polyhydramnios and congenital hypomyelinating neuropathy, along with severe respiratory insufficiency often requiring tracheostomy, and developmental delay. Survival beyond early infancy is almost uncommon. However, early extensive medical care has recently proven to elongate life expectancy, up to late childhood. Treatments have yet to be developed.

## Figures and Tables

**Table 1:** Review of clinical features associated with LCCS7 and CHN3.

Clinical feature		Percentage of patients		
		LCCS7	CHN3	Total
Prenatal manifestations	Preterm	26% (*n* = 8)	32% (*n* = 10)	58% (*n* = 18)
	Polyhydramnios	36% (*n* = 11)	61% (*n* = 19)	97% (*n* = 30)
	Reduced fetal movements (fetal akinesia)	22% (*n* = 7)	20% (*n* = 6)	42% (*n* = 13)

Head and neck	Microcephaly	0	10% (*n* = 3)	10% (*n* = 3)
	Dolichocephaly	3% (*n* = 1)	3% (*n* = 1)	6% (*n* = 2)
	Facial diplegia	16% (*n* = 5)	42% (*n* = 13)	58% (*n* = 18)
	Myopathic facial features	10% (*n* = 3)	13% (*n* = 4)	23% (*n* = 7)
	Head titubation	0	3% (*n* = 1)	3% (*n* = 1)
	Micrognathia	0	16% (*n* = 5)	16% (*n* = 5)
	Retrognathism	0	3% (*n* = 1)	3% (*n* = 1)
	Stiff jaw	3% (*n* = 1)	0	3% (*n* = 1)

Ophthalmologic	Ptosis	0	6% (*n* = 2)	6% (*n* = 2)
	Epicanthic folds	3% (*n* = 1)	3% (*n* = 1)	6% (*n* = 2)
	Nystagmus	0	3% (*n* = 1)	3% (*n* = 1)
	Anterior subcapsular cataract	3% (*n* = 1)	0	3% (*n* = 1)
	Microphthalmia	3% (*n* = 1)	3% (*n* = 1)	6% (*n* = 2)
	Vision loss	0	6% (*n* = 2)	6% (*n* = 2)

Ear, nose, throat	Sensorineural hearing loss	0	10% (*n* = 3)	10% (*n* = 3)
	Low-set ears	0	10% (*n* = 3)	10% (*n* = 3)
	Thickened nares	3% (*n* = 1)	6% (*n* = 2)	10% (*n* = 3)
	Thickened lips	3% (*n* = 1)	6% (*n* = 2)	10% (*n* = 3)
	Cleft palate	0	16% (*n* = 5)	16% (*n* = 5)
	Narrow ridged palate	0	29% (*n* = 9)	29% (*n* = 9)
	Thickened gums	0	13% (*n* = 4)	13% (*n* = 4)
	Extra teeth	0	3% (*n* = 1)	3% (*n* = 1)
	Notch in the upper gum midline	0	6% (*n* = 2)	6% (*n* = 2)
Respiratory	Respiratory distress	36% (*n* = 11)	64% (*n* = 20)	100% (*n* = 31)
Cardiac	Resting tachycardia	0	3% (*n* = 1)	3% (*n* = 1)
Gastrointestinal	Absent swallowing	36% (*n* = 11)	22% (*n* = 7)	58% (*n* = 18)

Musculoskeletal	Distal AMC	26% (*n* = 8)	16% (*n* = 5)	42% (*n* = 13)
	Multiple distal joint contractures	3% (*n* = 1)	6% (*n* = 2)	10% (*n* = 3)
	Flexion contracture of knees	0	6% (*n* = 2)	6% (*n* = 2)
	Flexion contracture of hands	3% (*n* = 1)	10% (*n* = 3)	13% (*n* = 4)
	Flexion contracture of elbows	0	13% (*n* = 4)	13% (*n* = 4)
	Flexion contracture of fingers	0	29% (*n* = 9)	29% (*n* = 9)
	Flexion contracture ankle	0	6% (*n* = 2)	6% (*n* = 2)
	Flexion posture with ulnar deviation of the wrist	0	3% (*n* = 1)	3% (*n* = 1)
	Torticollis	3% (*n* = 1)	0	3% (*n* = 1)
	Partial bilateral toe syndactyly	0	6% (*n* = 2)	6% (*n* = 2)
	Foot varus deformity	0	6% (*n* = 2)	6% (*n* = 2)
	Clubfeet	29% (*n* = 9)	10% (*n* = 3)	39% (*n* = 12)

Neurologic	Hypotonia	36% (*n* = 11)	51% (*n* = 16)	87% (*n* = 27)
	Areflexia	22% (*n* = 7)	22% (*n* = 7)	45% (*n* = 14)
	Increased reflexes	0	6% (*n* = 2)	6% (*n* = 2)
	Positive Babinski sign	0	6% (*n* = 2)	6% (*n* = 2)
	Ataxia	0	3% (*n* = 1)	3% (*n* = 1)
	Tremor	0	3% (*n* = 1)	3% (*n* = 1)
	Dystonia	0	6% (*n* = 2)	6% (*n* = 2)
	Reduced gesticulation	0	6% (*n* = 2)	6% (*n* = 2)
	Generalized epilepsy (tonic-clonic seizures)	3% (*n* = 1)	10% (*n* = 3)	13% (*n* = 4)
	Myoclonic seizures	0	6% (*n* = 2)	6% (*n* = 2)

Developmental	Developmental disabilities	0	29% (*n* = 9)	29% (*n* = 9)
	Intellectual disability	0	29% (*n* = 9)	29% (*n* = 9)

Dermatologic	Small nails	0	3% (*n* = 1)	3% (*n* = 1)
	Naevus flammeus	0	3% (*n* = 1)	3% (*n* = 1)
	Eczema	0	3% (*n* = 1)	3% (*n* = 1)
	Abnormal skin pigmentation	0	3% (*n* = 1)	3% (*n* = 1)

Urogenital	Undescended testes	0	3% (*n* = 1)	3% (*n* = 1)
	Neurogenic bladder	0	6% (*n* = 2)	6% (*n* = 2)

AMC: arthrogryposis multiplex congenita; LCCS7: lethal congenital contracture syndrome type 7; CHN3: congenital hypomyelinating neuropathy type 3.

**Table 2:** Variants in *CNTNAP1* gene.

Patient	Ethnicity	Age at death	Sex	Variant	Type	Exon	Transmission	Disease
1 [1]	Palestinian	Is now 9 yo	M	c.2015G>A:p.(Trp672^*∗*^)	Nonsense	13	Homozygous	CHN3
2 [1]	Palestinian	Is now 12 yo	M	c.2015G>A:p.(Trp672^*∗*^)	Nonsense	13	Homozygous	CHN3
3 [1]	Palestinian	Is now 4 w	M	c.2015G>A:p.(Trp672^*∗*^)	Nonsense	13	Homozygous	CHN3
4 [1]	Northern Irish	4 m	M	c.2011C.T:p.(Gln671^*∗*^)	Nonsense		Heterozygous	CHN3
				c.2290C.T:p.(Arg764Cys)	Missense			
5 [1]	Northern Irish	4 h	M	c.2011C.T:p.(Gln671^*∗*^)	Nonsense		Heterozygous	CHN3
				c.2290C.T:p.(Arg764Cys)	Missense			
6 [2]	French	4 h	M	c.2289C>T:p.(Arg764Cys)	Missense		Heterozygous	CHN3
				c.2011C>T:p.(Gln671^*∗*^)	Nonsense			
7 [2]	French	2 m	M	c.967T>C:p.(Cys323Arg)	Missense	7	Heterozygous	CHN3
				c.1869G>A:p.(Trp623^*∗*^)	Nonsense	13		
8 [2]	French	1 h	M	c.967T>C:p.(Cys323Arg)	Missense	7	Heterozygous	CHN3
				c.1869G>A:p.(Trp623^*∗*^)	Nonsense	13		
9 [8]	Qatari	Is now 13 yo	F	c.1561dupC:p.(Leu521ProfsX12)	Nonsense		Homozygous	LCCS7
10 [8]	Qatari	1 h	F	c.1561dupC:p.(Leu521ProfsX12)	Nonsense		Homozygous	LCCS7
11 [8]	Qatari	1 h	F	c.1561dupC:p.(Leu521ProfsX12)	Nonsense		Homozygous	LCCS7
12 [6]	French	10 d	M	c.2901_2902del (P967PfsX12)	Frameshift	18	Homozygous	LCCS7
13 [6]	French	10 d	M	c.2901_2902del (P967PfsX12)	Frameshift	18	Homozygous	LCCS7
14 [6]	French	10 d	M	c.2901_2902del (P967PfsX12)	Frameshift	18	Homozygous	LCCS7
15 [6]	French	1 m	M	c.3009_3010insT (F1003fs)	Frameshift	19	Homozygous	LCCS7
16 [6]	French	1 m	M	c.3009_3010insT (F1003fs)	Frameshift	19	Homozygous	LCCS7
17 [6]	French	1 m	M	c.3009_3010insT (F1003fs)	Frameshift	19	Homozygous	LCCS7
18 [6]	French	10 d	M	c.2993-2_2994del (I999WfsX5)	Frameshift	19	Homozygous	LCCS7
19 [7]	American	1 m	M	c.1163G>C:p.(Arg388Pro)	Missense		Homozygous	CHN3
20 [10]	English	Is now 15 yo	F	c.2141G>C:p.(Arg714Pro)	Missense		Homozygous	CHN3
21 [10]	English	Is now 8 yo	M	c.635T>C:p.(Leu212Pro)	Missense		Heterozygous	CHN3
				c.1677G>A:p.(Trp559^*∗*^)	Nonsense			CHN3
22 [10]	English	Is now 6 yo	M	c.635T>C:p.(Leu212Pro)	Missense		Heterozygous	CHN3
				c.1677G>A:p.(Trp559^*∗*^)	Nonsense			
23 [10]	English	Is now 7 yo	M	c.2344C>T:p.(Arg782^*∗*^)	Nonsense		Homozygous	CHN3
24 [10]	English	Is now 4 yo	F	c.1735+1G>A	Nonsense		Heterozygous	CHN3
				c.2344C>T:p.(Arg782^*∗*^)	Nonsense			
25 [10]	English	Is now 2 yo	M	c.149C>A:p.(Pro50Gln)	Missense		Heterozygous	CHN3
				c.2600del:p.(Asp867fs)	Frameshift			
26 [10]	English	3 m	F	c.1861C>T:p.(Arg621^*∗*^)	Nonsense		Heterozygous	CHN3
				c.2687G>A:p.(Trp896^*∗*^)	Nonsense			
27 [11]	French	2 m	M	c.967T4C:p.(Cys323Arg)	Missense	7	Heterozygous	CHN3
				c.1869G4A:p.(Trp623^*∗*^)	Nonsense	13		
28 [11]	French	1 h	M	c.967T4C:p.(Cys323Arg)	Missense	7	Heterozygous	CHN3
				c.1869G4A:p.(Trp623^*∗*^)	Nonsense	13		
29 [9]	American	Is now 8 yo	M	c.1163G>C: p.(Arg388Pro)			Homozygous	CHN3
30 [9]	American	8 y	F	c.967T>C: p.(Cys323Arg)	Missense	7	Heterozygous	CHN3
				c.319C>T:p.(Arg107^*∗*^)				
31	Lebanese	Is now 7 yo	M	c.3361C>T: p.(Arg1121^*∗*^)	Nonsense		Homozygous	LCCS7
